# Supplementary material for: Complexome profiling of the Chlamydomonas psb28 mutant reveals TEF5 as an early PSII assembly factor
Source: Plant Cell. 2025 Mar 17;37(6):koaf055. doi: 10.1093/plcell/koaf055 (PMC12164590; doi:10.1093/plcell/koaf055)
Supplement: koaf055_Supplementary_Data [file koaf055_supplementary_data.zip › psb28_interactive.zip!/plots/intensity/qualityPlots/peptides/_Cre16.g660150.t1.2.html]

### Cre16.g660150.t1.2

Trivial name:   
SeqCoverage [median]: 19.80  
Mass: 13.31 kDa  
  
 

| Sequence | MissedCleavage | Mass [Da] | wtIntensity | muIntensity | Score |
| --- | --- | --- | --- | --- | --- |
| AGVEAEAPQVHR | 0 | 1262.64 | 714.57 | 871.40 | 72.65 |
| LIDLHSPADVVK | 0 | 1305.73 | 477.49 | 0.00 | 84.66 |
|  |  |  | 1192.1 | 871.4 |  |
